# Psychometric analysis of the patient-reported outcomes measurement information system parent proxy physical function–upper extremity item bank for children with Duchenne muscular dystrophy

**DOI:** 10.3389/fneur.2025.1481825

**Published:** 2025-05-13

**Authors:** Linda Pax Lowes, Corinne M. Le Reun, Teofil Ciobanu, Lindsay N. Alfano, Natalie F. Reash, Megan A. Iammarino, Shivangi Patel, Ivana F. Audhya

**Affiliations:** ^1^Center for Gene Therapy, Abigail Wexner Research Institute at Nationwide Children’s Hospital, Columbus, OH, United States; ^2^Biostatistics, Sainte-Anne, Guadeloupe, France; ^3^F. Hoffmann-La Roche Ltd., Basel, Switzerland; ^4^Patient-Centered Outcomes, Sarepta Therapeutics, Inc., Cambridge, MA, United States

**Keywords:** disability, neuromuscular, patient-reported outcome measures, psychometrics, quality of life, validity

## Abstract

**Background:**

The Patient-Reported Outcomes Measurement Information System (PROMIS) is a collection of generic patient-reported outcome instruments used to quantify disease impact on a variety of functional subdomains, including physical, cognitive, emotional, and other domains. The reliability and validity of the PROMIS Parent Proxy (PP) Physical Function–Upper Extremity (UE) item bank is not established in children with Duchenne muscular dystrophy (DMD). This study evaluated the psychometric properties and measurement quality of the PROMIS PP UE item bank v1.0 (29 items) in DMD using a Rasch psychometric analysis.

**Methods:**

The PROMIS PP UE item bank was completed by caregivers of children with DMD aged at least 8 years, under care at Nationwide Children’s Hospital (Columbus, OH, United States). Rasch analysis was used to evaluate the psychometric performance of the measure and its items in DMD, based on several criteria, including item–trait interaction, individual items fit, Person Separation Index (PSI), individual persons fit, and response dependency.

**Results:**

Rasch analysis was conducted on 206 observations. Several items had weak clinical utility in measuring upper extremity functioning in DMD. Additionally, the analysis identified specific response options that could be restructured to improve the reliability and precision of the items in evaluating upper extremity function in DMD. A new customized 21-item measure demonstrated overall good fit to Rasch model expectations (*p* = 0.095; nonsignificant) and the ability to discriminate among respondents with different levels of upper extremity function (0.95 PSI; excellent reliability). Upper extremity function was generally well targeted across the severity spectrum, except for the least severe patients.

**Conclusion:**

The customized PROMIS PP UE measure conformed to Rasch assumptions, indicating that it can serve as a reliable option for caregiver-reported upper extremity assessment in DMD.

## 1 Introduction

Duchenne muscular dystrophy (DMD) is a rare genetic neuromuscular disease characterized by progressive muscle degeneration, resulting in weakness and loss of physical function (1–3). DMD is inherited in an X-linked recessive pattern and primarily affects males (4, 5). The global prevalence of DMD is 7.1 per 100,000 males, and it has been estimated that there are between 9,000 and 12,000 males with DMD in the United States (4, 6). Typically, DMD symptoms appear in early childhood and include muscle weakness that leads to frequent falls and difficulty with motor skills, such as climbing stairs and getting up from the floor. Progressive muscle weakness leads to complete loss of ambulation by the teenage years (1, 7). Upper limb function is also affected. Caregivers frequently report difficulties with fine motor skills early in the disease (8). The decline of upper limb function can manifest when the child is still ambulatory, and the loss of upper limb function is known to represent a challenging transition for both patients and their families as it directly affects patients’ abilities to carry out essential activities of daily living (ADLs) independently. ADLs encompass routine tasks such as dressing, grooming, eating, and performing personal hygiene. This shift can significantly impact their overall quality of life and sense of autonomy (5, 8, 9).

Patient-reported outcomes (PROs) and caregiver proxy or observer–reported outcomes (ObsROs) are measures that are used to assess function outside of the clinic, providing valuable information about the impact of a disease on a patient’s daily life (10–12). Both PROs and ObsROs provide unique and important information about the patient’s functional abilities. When a child is too young or incapable of completing the assessment, caregivers can act as a proxy to report on their care recipient’s observable symptoms. When it comes to assessing PROs, the perspectives of patients and their caregivers can sometimes diverge. However, using ObsROs helps maintain consistency in data collection over extended timeframes, even in clinical trials in which patients may age up during the study. Thus, the perspectives of caregivers hold significant value because they contribute to a more comprehensive understanding of the patient’s condition (13).

The impact of DMD on health-related quality of life has been assessed using several PRO and ObsRO measures, including generic preference-based measures, such as the EQ-5D and Pediatric Quality of Life Inventory (PedsQL) Generic Core, and condition-specific measures, such as the PedsQL DMD module and DMD-QoL (3, 14–16). Yet, the validity of these and other measures in children with DMD is not well established (3).

The Patient-Reported Outcomes Measurement Information System (PROMIS) (see https://www.promishealth.org/57461-2/ and https://commonfund.nih.gov/promis/index) represents a set of generic PRO questionnaires designed to evaluate various aspects of health-related quality of life, including physical, mental, and social functioning, across different populations and health conditions. The generic PROMIS Parent Proxy (PP) Physical Function–Upper Extremity (UE) item bank was specifically developed to measure caregivers’ perceptions of a child’s upper extremity function, such as activities that require use of the shoulders, arms, and hands, over the week prior to the completion of the item bank.^1^ The PROMIS PP UE and other PROMIS measures have been used in patients with DMD at Nationwide Children’s Hospital’s Neuromuscular Clinic during clinical practice, as well as in other studies (11, 17–19). While these measures offer standardized tools for the evaluation of outcomes, they require validation in a specific population of interest to ensure their relevance and accuracy. Validating PROMIS measures in the context of DMD entails evaluating the measure’s performance and suitability specifically for this patient population. Since the psychometric validity of PROMIS PP UE in measuring upper body function in children with DMD has not been established, further psychometric testing of the PROMIS PP UE item bank is warranted.

The present study therefore aimed to evaluate the validity and psychometric performance of the generic PROMIS PP UE 29-item bank in a cohort of children with DMD through Rasch statistical analysis. Validation of ObsRO measures is important in DMD, as no gold standard ObsRO measure exists to date.

## 2 Methods

### 2.1 Data collection and study population

All caregivers of ambulatory and non-ambulatory male patients aged 8 years and older with genetically confirmed DMD were asked to complete the item bank as part of their clinical care at the Neuromuscular Clinic at Nationwide Children’s Hospital (Columbus, OH, United States). Because patients aged up to 8 years are still developing gross and fine motor skills and acquiring upper extremity function, they were excluded from this analysis to avoid bias in the assessment of functional ability in this population. Given the documented tendency of children, especially those with DMD, to overestimate their abilities and the resulting strong ceiling effect in self-reports, we opted to rely on caregiver/proxy reporting for assessing children’s upper extremity function, despite including children old enough to self-report in our sample (13, 20–22).

Deidentified PROMIS data were transferred to an independent biostatistician, who utilized all available PROMIS records at the time of transfer to conduct this psychometric analysis. An institutional review board (IRB) waiver was obtained before conducting this analysis.

### 2.2 Study item bank

Caregivers completed the generic PROMIS PP UE version 1.0 item bank (29 items; access to the item bank along with scoring and interpretation instructions are found at HealthMeasures.net). The initial 27 items of the item bank represent actions that the child may or may not be capable of executing, and these items are evaluated using a categorical scoring system ranging from 0 (not able to do) to 4 (with no trouble) (Table 1). The last 2 items inquire about the need for assistance or special equipment for specific tasks, with responses ranging from 0 (almost always) to 4 (never).

**Table 1:** Ability scoring for the first 27 items.

Score	Response options
4	With no trouble
3	With a little trouble
2	With some trouble
1	With a lot of trouble
0	Not able to do

### 2.3 Approach

This analysis is based on Rasch Measurement Theory, a family of statistical models used to assess internal functioning of instruments and items. The Rasch model is a method of mapping a rating scale against a mathematical measurement model using person-level responses to individual items to estimate their position on a continuum of a latent trait (here, upper extremity function). The model then statistically evaluates the assumption that respondents with higher levels of the construct (e.g., better upper extremity function) select higher response categories, while respondents with lower levels of the construct (e.g., worse upper extremity function) select lower response categories. The Rasch model provides information on how well items of the scale work to measure the latent trait, which can then be used to refine the instrument to accurately measure and differentiate between the range of abilities specific to a population of interest (23). A schematic representation of the Rasch analysis conducted in this study is provided in Figure S1.

### 2.4 Statistical analysis

Psychometric evaluation was completed by fitting a Rasch partial credit model with 3 class intervals using RUMM2030 software (RUMM Laboratory; Perth, Australia) (24). The Rasch model was implemented iteratively, requiring revisions to meet the requirement that the model appropriately order response options to each item in ascending order of difficulty, in accordance with their definitions. Therefore, guided by category probability curves and clinical soundness of regrouping levels of responses, revisions included assessing category thresholds and collapsing neighboring response categories as needed. In accordance with Rasch methodology, the model was rerun until all items had ordered thresholds.

Performance of the PROMIS PP UE item bank was examined in the following areas: item-trait interaction, individual items and persons fit, Person Separation Index (PSI), and response dependency. The data fit was tested against Rasch model expectations through item-trait interaction, with a nonsignificant *p* value (*p* > 0.05) interpreted as evidence of model fit. Individual item fit (i.e., assessing whether each item fits the Rasch expectations) and person fit (i.e., understanding whether each person’s responses to the various items aligns with their abilities, as expected by the Rasch model) were also assessed. To demonstrate adequate model fit for individual item and person fit, residual values ideally fall between −2.5 and 2.5, and, for individual item fit, a chi-square *p* value should be nonsignificant after adjusting for multiple comparisons using the Bonferroni method. The PSI indicates how effectively the assessment tool differentiates between respondents with varying levels of the trait being measured, in this case varying levels of upper extremity function; values higher than 0.7, 0.8, and 0.9 indicate acceptable, good, and excellent reliability, respectively. The residual correlation matrix was used to assess local dependency (a situation in which a response to 1 item influences a response to another). Correlation values above 0.3 were seen as potentially indicative of local dependency and corresponding items were reviewed, taking their clinical relevance into consideration. Unidimensionality was tested through principal component analysis of the residuals using the method proposed by Smith (25). The correlations between items and the first residual factor were examined to produce separate person estimates based on two subsets of items (items positively vs. negatively correlated to the first factor). By using an independent t-test for the difference in these estimates for each person, the percentage of tests falling outside the range −1.96 to 1.96 should not exceed 5%, i.e., the associated binomial confidence interval should overlap the 5% expected value for the scale to be considered unidimensional (26, 27).

Differential item functioning (DIF) occurs when individuals from different groups, with the same level of ability on the latent trait being measured, score differently on some items. DIF is assessed by evaluating each item for signs of interactions with sample characteristics. In this analysis, the participant’s ambulatory status, recorded at the time the item bank was administered at the clinic, is the only variable explored as a potential source of DIF. In RUMM2030, two types of DIF are tested through analysis of variance models: (1) uniform DIF, representing different responses according to the group variable (i.e., a “shift” of the item on the underlying UE continuum, depending on which group the participants belong to) and (2) non-uniform DIF, representing an interaction between the item and the group variable (i.e., a difference in the way the item discriminates between participants, depending upon which group they belong).

## 3 Results

### 3.1 Preliminary analysis

In total, 206 observations were entered into the RUMM software for Rasch analysis. The average age in this sample was 12.4 years (standard deviation [SD], 3.0; range 8–27) with 41% of observations recorded while children were still ambulatory. According to the clinical judgment of neuromuscular physical therapists at Nationwide Children’s Hospital, a decision was made to not incorporate 4 items from the item bank into the Rasch analysis because these items were considered irrelevant for measuring upper extremity function in children with DMD. These 4 items were: “ability to tie shoelaces without help,” “ability to dial a phone,” “ability to hold an empty cup,” and “ability to move hands or fingers.” The skill of independently tying shoelaces was omitted owing to clinical observation indicating that children with DMD seldom wear shoes with shoelaces. Based on observations that parents had difficulty understanding the item “ability to dial a phone,” and owing to a perceived item obsolescence in the contemporary context, this item was also considered not relevant. The exclusion of the “ability to hold an empty cup” and “ability to move hands or fingers” was attributed to these abilities not being impacted by DMD until the end of the disease progression, at which point it would be highly unlikely that the PROMIS PP UE item bank would be administered.

**Figure 1:**
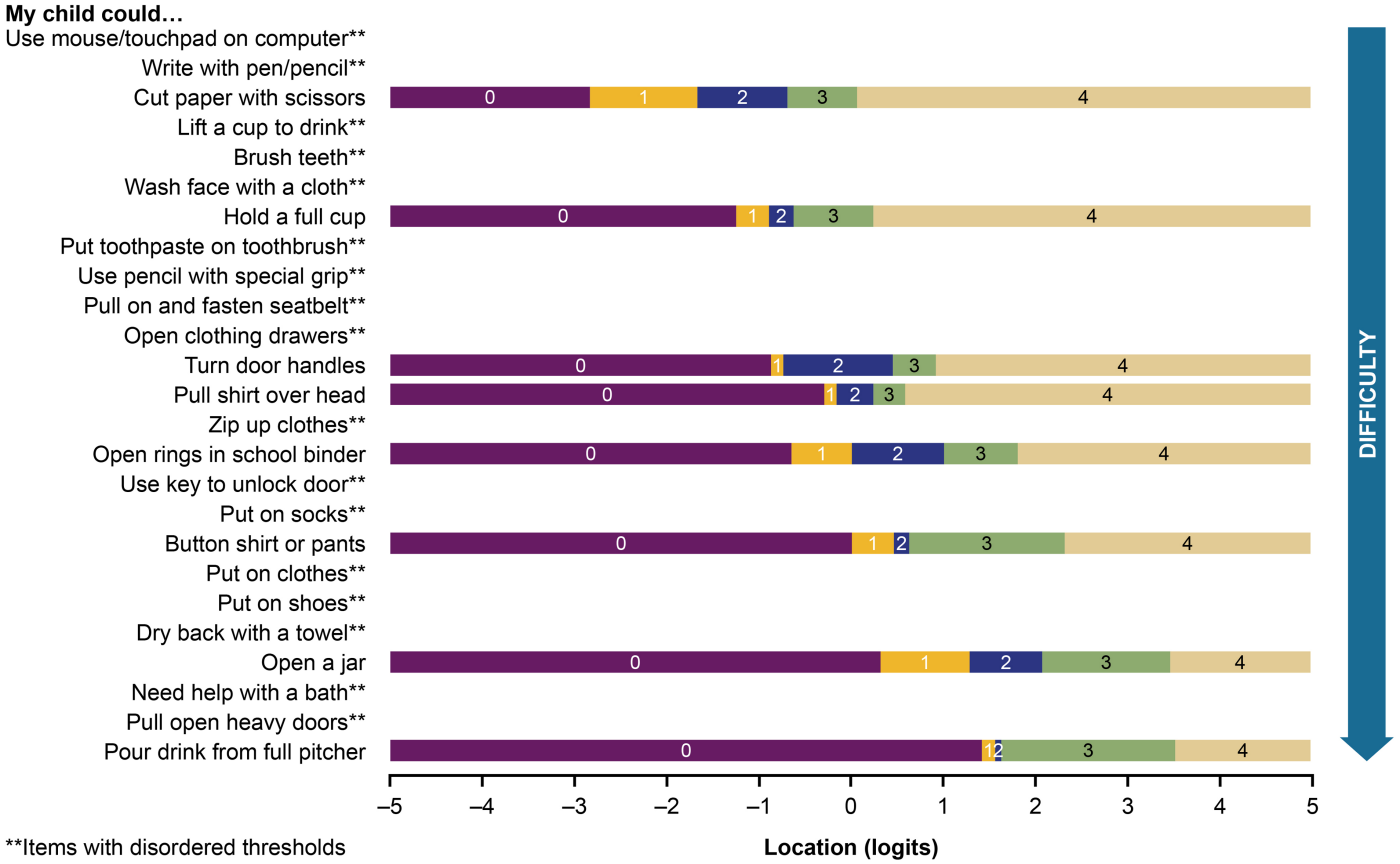
Threshold map for the initial 25-item analysis of the PROMIS parent proxy – upper extremity item bank. The labels 0 through 4 represent the 5 original response options, ordered by level of difficulty (0 = “not able to do,” 1 = “with a lot of trouble,” 2 = “with some trouble,” 3 = “with a little trouble,” and 4 = “with no trouble”). Recall period was the past 7 days.

Rasch analysis was therefore conducted on 25 items from the item bank. The first model iteration did not provide a satisfactory fit to the Rasch assumptions. The item-trait interaction was associated with a significant *p* value (*p* < 0.001, χ^2^ value 165.9 with 50 degrees of freedom), and there were signs of item misfits. Only 8 of the 25 items had appropriately ordered thresholds, suggesting that the proposed scoring system did not work as intended for the remaining 17 items (Figure 1).

### 3.2 Reorganization of response categories and changes to model

**Figure 2:**
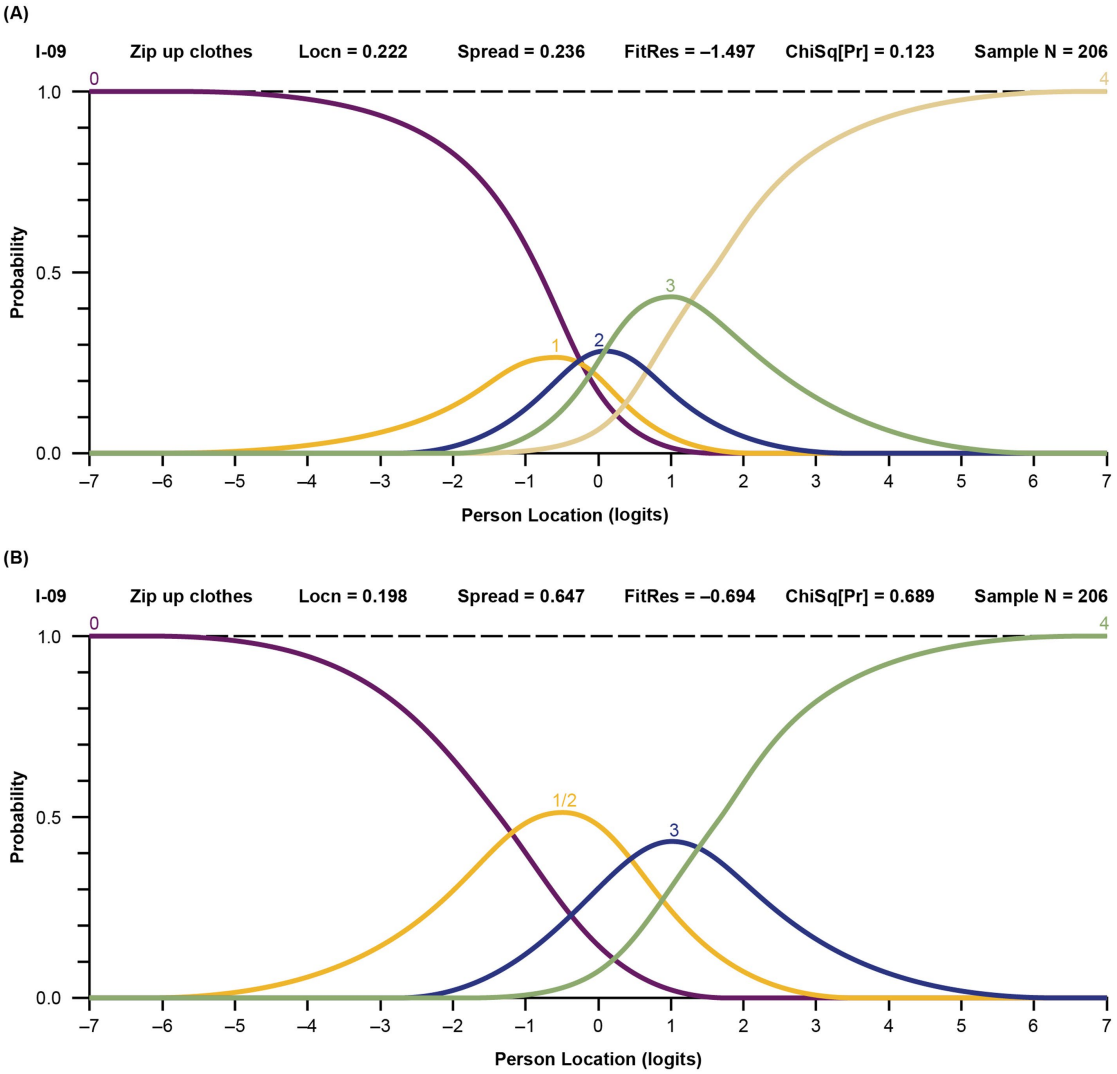
Probability curves for disordered item 9, “z*ip up clothes*,” before **(A)** and after **(B)** thresholds regrouping. **(A)** Before regrouping. The labels 0 through 4 represent the 5 original response options (0 = “not able to do,” 1 = “with a lot of trouble,” 2 = “with some trouble,” 3 = “with a little trouble,” and 4 = “with no trouble”). The yellow line indicates the item (1 = “with a lot of trouble”) that was flagged in the model as having disordered threshold. **(B)** After regrouping. The labels 0 through 4 now represent the adjusted response options (0/1 = “not able to do” and “with a lot of trouble” grouped together, 2 = “with some trouble,” 3 = “with a little trouble,” and 4 = “with no trouble”). Locn, location; FitRes, fit residual; ChSq(Pr), chi square probability.

**Figure 3:**
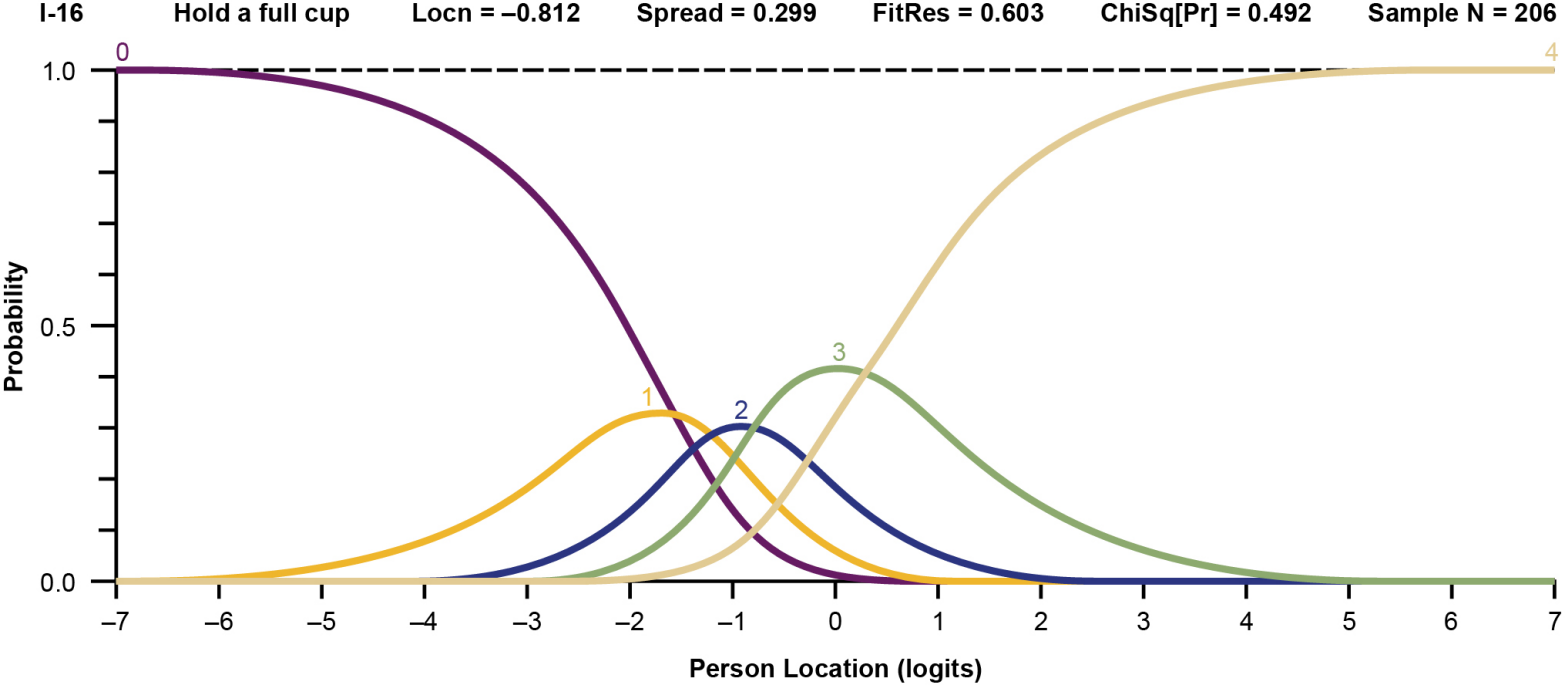
Probability curves for the ordered item 16, “able to hold a full cup,” in the final model. The labels 0 through 4 represent the 5 original response options (0 = “not able to do,” 1 = “with a lot of trouble,” 2 = “with some trouble,” 3 = “with a little trouble,” and 4 = “with no trouble”). Locn, location; FitRes, fit residual; ChSq(Pr), chi square probability.

Response categories were reorganized for items with disordered thresholds, based on observation of the probability curves and clinical input. An example of probability curves for an initially disordered item is item 9 (“zip up clothes”); before and after regrouping thresholds are shown in Figure 2. In this scenario, response option 1 (“with a lot of trouble”) posed problems and was rarely selected, indicating that the respondents did not perceive it clearly. Consequently, item 9 exhibited disordered thresholds. To address this issue, response option 1 was merged with response option 2 (“with some trouble”), based on expert clinician opinion. A similar approach was applied to all items with disordered thresholds, and the model was re-run using the new categories. An example of probability curves for an item with 5 ordered and distinct curves based on the initial 5 response categories is item 16 (“able to hold a full cup”) (Figure 3).

After all thresholds were ordered for all items, the item trait interaction *p* value remained significant, indicating that the model still did not satisfactorily fit the Rasch assumptions. Item content was reevaluated, and quantitative and qualitative evidence appraised to inform subsequent decisions. The residual correlation matrix revealed some relatively high levels of correlation among a few items. Considering the presence of multiple items capturing similar tasks and functions, the decision was made to exclude three: “ability to open rings in a school binder,” “ability to put on shoes without help,” and “ability to put on clothes without help.” In addition, the item “need for a pencil with special grip to write” was identified as not relevant, as pencils with a special grip are not consistently used by children with DMD. Therefore, the Rasch analysis was restricted to the remaining 21 items.

**Figure 4:**
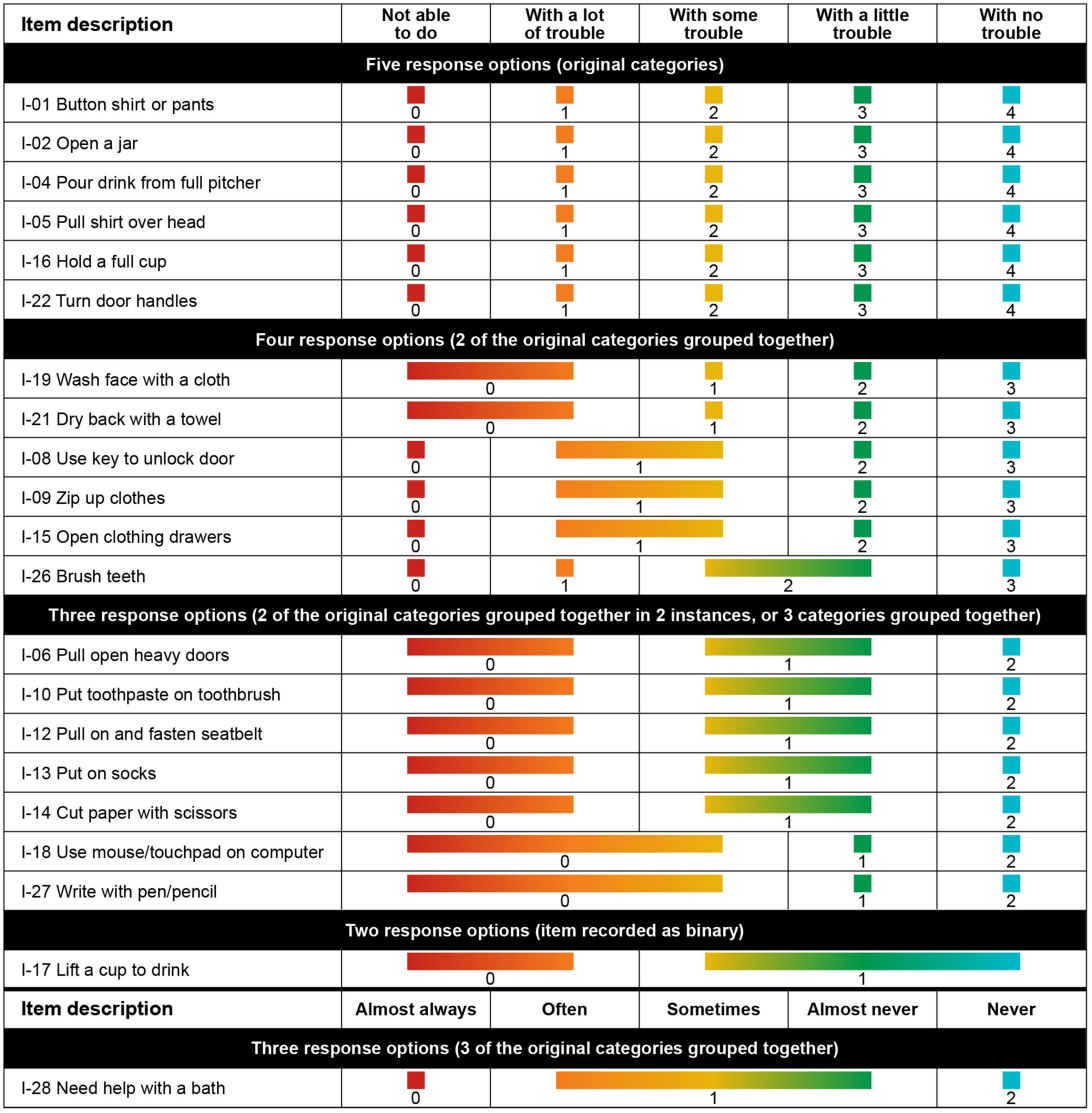
Best clinical and mathematical fit, achieved after rescoring response options for 12 items.

Response categories were reorganized for 15 of the 21 items in the finalized model. Six items had their response options collapsed from the original 5 levels to 4, 8 items had their response options collapsed from the original 5 levels to 3, and 1 item had its response options collapsed from the original 5 levels to 2 (Figure 4). The remaining 6 items were retained without modification to optimize the granularity of the most clinically relevant items for upper extremity function and daily activities.

### 3.3 Finalized Rasch results

#### 3.3.1 Overall model fit

The finalized Rasch model was associated with a nonsignificant item-trait interaction *p* value (*p* = 0.095, χ^2^ value 54.4 with 42 degrees of freedom), indicating a good overall model fit to the data. The ability of the item bank to distinguish among respondents with varying degrees of upper extremity function was assessed as excellent (PSI, 0.95). Every item and every person fit the expectations of the Rasch model adequately. In accordance with the target values of 0 (SD, 1.00), the average item and corresponding person fit residual values (SD) were −0.22 (1.09) and −0.23 (0.76), respectively. Unidimensionality was satisfied.

#### 3.3.2 Item locations

Item locations and associated fit values are presented in Table 2. Items are ordered according to the level of difficulty with which they are associated on the underlying upper extremity function continuum, from easiest to most difficult. As expected for a population of late ambulatory and non-ambulatory pediatric children with DMD, the easiest items included “ability to lift a cup to drink” and “use a mouse or touchpad on a computer.” The most challenging activities were “pulling open heavy doors,” “taking a bath without help,” and “pouring a drink from a full pitcher”.

**Table 2:** Finalized item fit in measuring upper extremity function using PROMIS items in DMD.

Item number	Item description	Location	SE	Fit residual	*P* value
I-17	Lift a cup to drink	−3.80	0.43	−0.40	0.763
I-18	Use mouse/touchpad on computer	−3.29	0.26	−0.10	0.684
I-26	Brush teeth	−1.79	0.14	−0.39	0.703
I-19	Wash face with a cloth	−1.46	0.12	−0.23	0.220
I-27	Write with pen/pencil	−1.43	0.16	0.91	0.082
I-14	Cut paper with scissors	−1.23	0.15	0.95	0.613
I-16	Hold a full cup	−0.81	0.10	0.60	0.492
I-10	Put toothpaste on toothbrush	−0.67	0.14	−1.92	0.071
I-15	Open clothing drawers	−0.19	0.11	−0.34	0.231
I-22	Turn door handles	−0.14	0.09	0.86	0.509
I-05	Pull shirt over head	0.01	0.08	−1.43	0.307
I-12	Pull on and fasten seatbelt	0.05	0.13	−1.81	0.084
I-09	Zip up clothes	0.20	0.10	−0.69	0.689
I-08	Use key to unlock door	0.83	0.10	1.35	0.108
I-01	Button shirt or pants	0.89	0.09	1.04	0.071
I-13	Put on socks	1.14	0.12	−1.81	0.148
I-02	Open a jar	1.93	0.09	1.56	0.655
I-21	Dry back with a towel	2.04	0.10	−0.73	0.076
I-04	Pour drink from full pitcher	2.25	0.09	−1.07	0.771
I-28	Need help with bath	2.44	0.14	0.39	0.509
I-06	Pull open heavy doors	3.03	0.15	−1.31	0.231

#### 3.3.3 Threshold map

**Figure 5:**
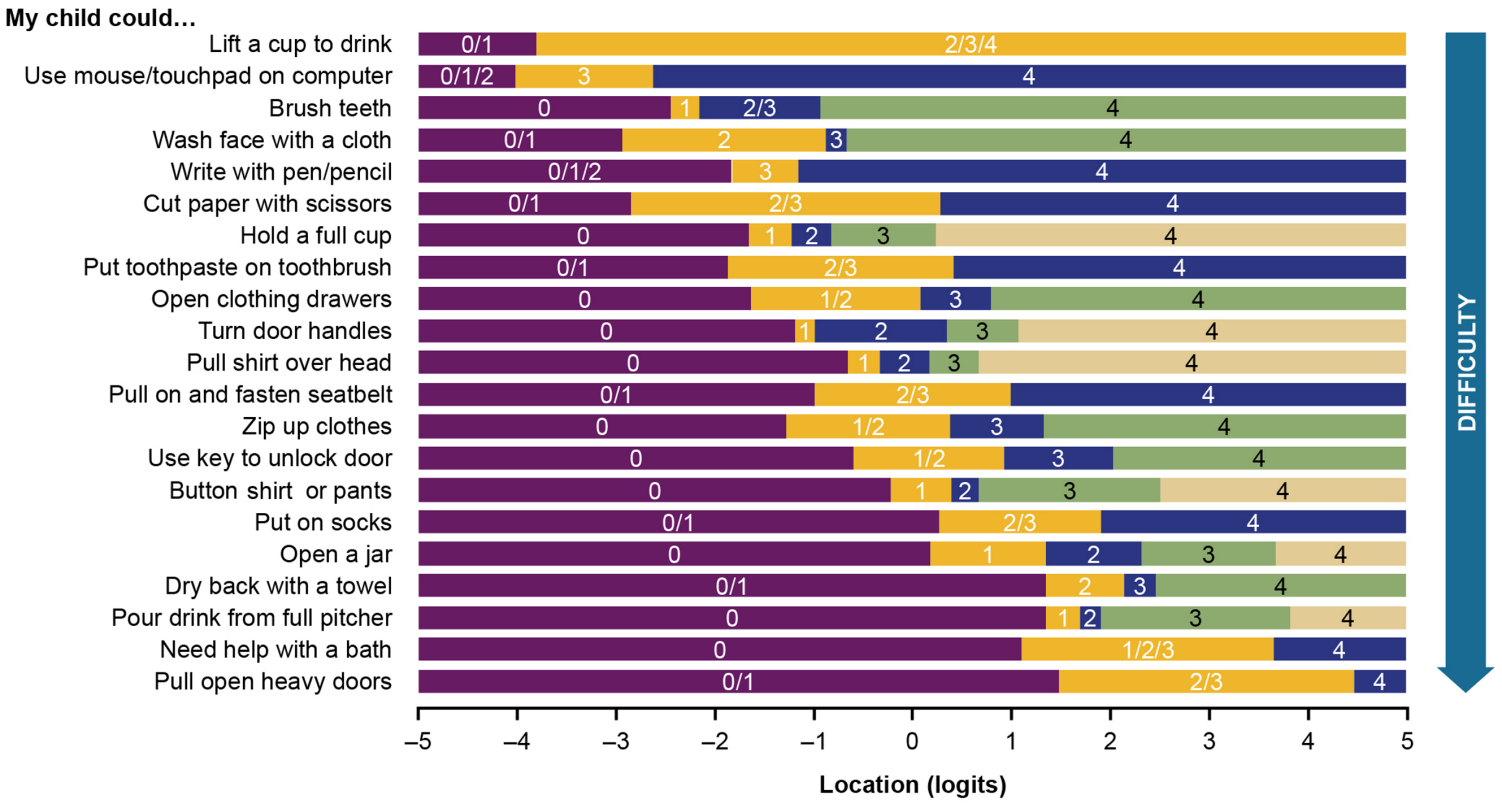
Threshold map for the customized PROMIS parent proxy – upper extremity item bank. The threshold map displays items ordered by level of difficulty and shows how the response options for each item are located on the underlying upper extremity function continuum. The labels 0–4 indicate the 5 original response options (0 = “not able to do,” 1 = “with a lot of trouble,” 2 = “with some trouble,” 3 = “with a little trouble,” and 4 = “with no trouble”). Some response options have been regrouped for certain items (e.g., 0/1 shows the first 2 response options merged).

The customized PROMIS PP UE measure threshold map, with items arranged according to the level of upper extremity difficulty, shows that there are now ordered thresholds for every item (Figure 5). The map demonstrates that loss of ability is not observed uniformly across items and that pediatric patients with DMD have an increasing level of difficulty performing activities as their overall upper extremity function gradually declines.

#### 3.3.4 Person-item locations

The average person-item location was 0.88 (range −4.09 to 4.50), indicating that patients performed, on average, slightly better on the upper extremity function continuum than expected by the model. There were no extreme values, therefore, no floor or ceiling effect.

#### 3.3.5 Person-item location and threshold distributions

**Figure 6:**
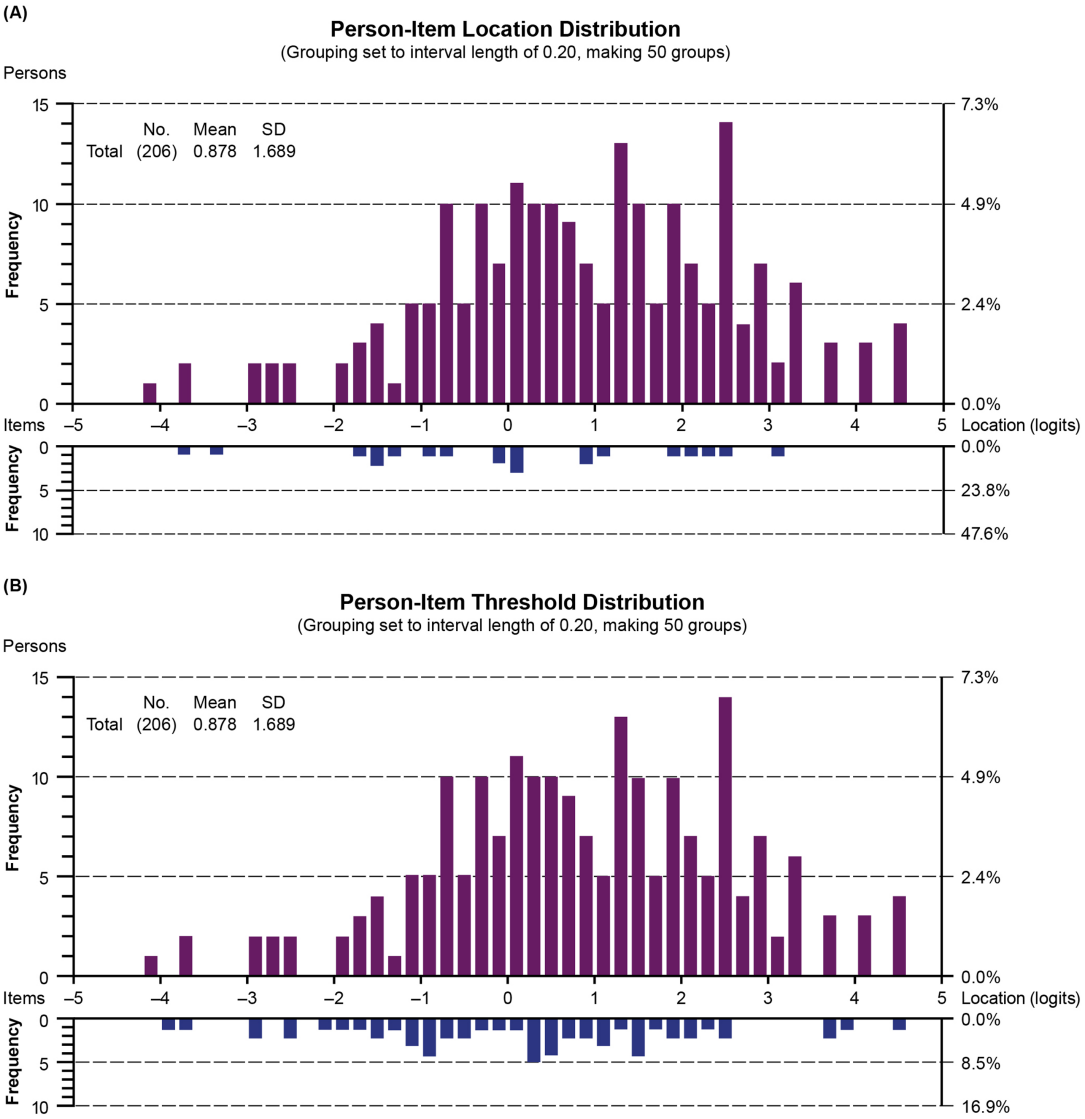
Person-item location **(A)** and threshold **(B)** distribution (grouping set to interval length of 0.20, making 50 groups). Children with DMD with the highest upper extremity level and the most difficult items are plotted on the right side of the histogram. Those with the lowest upper extremity level and the least difficult items are plotted on the left side. SD, standard deviation.

The person-item location and threshold distributions of the revised measure are shown in Figure 6, which illustrates person-item alignment, where person ability (i.e., level of upper extremity function) and item location (i.e., average difficulty level) and item thresholds (i.e., upper extremity function level where respondents move from one response level to the next) are plotted together. The item thresholds covered a wider range of the underlying upper extremity function continuum than the average item locations and were better matched to the respondent’s distribution. Overall, item locations are well spread out on the continuum, indicating good coverage across varying levels of upper extremity function. However, a noticeable gap in the histogram for location values greater than 3 and threshold values between 2.5 and 3.5 indicates that PROMIS items and thresholds may not be sufficiently targeting the higher level of upper extremity function. It may be beneficial to add more items to broaden the construct under study in order to differentiate respondents with DMD who exhibit high upper extremity function.

#### 3.3.6 Local dependency

Only 2 correlations between 0.3 and 0.4 in absolute value were observed—between item 13 (“my child could put on his socks without help”) and items 2 (“my child could open a jar by himself”) and 9 (“my child could zip up his clothes”). Given that these items were not highly correlated and address distinct aspects of the underlying construct, it was deemed reasonable to retain both. Their strong clinical relevance and effective performance within the context of other parameters support this decision. All other correlations observed were within the acceptable range of the model. The residual correlation matrix is shown in Table S1.

#### 3.3.7 Exploration of DIF

**Figure 7:**
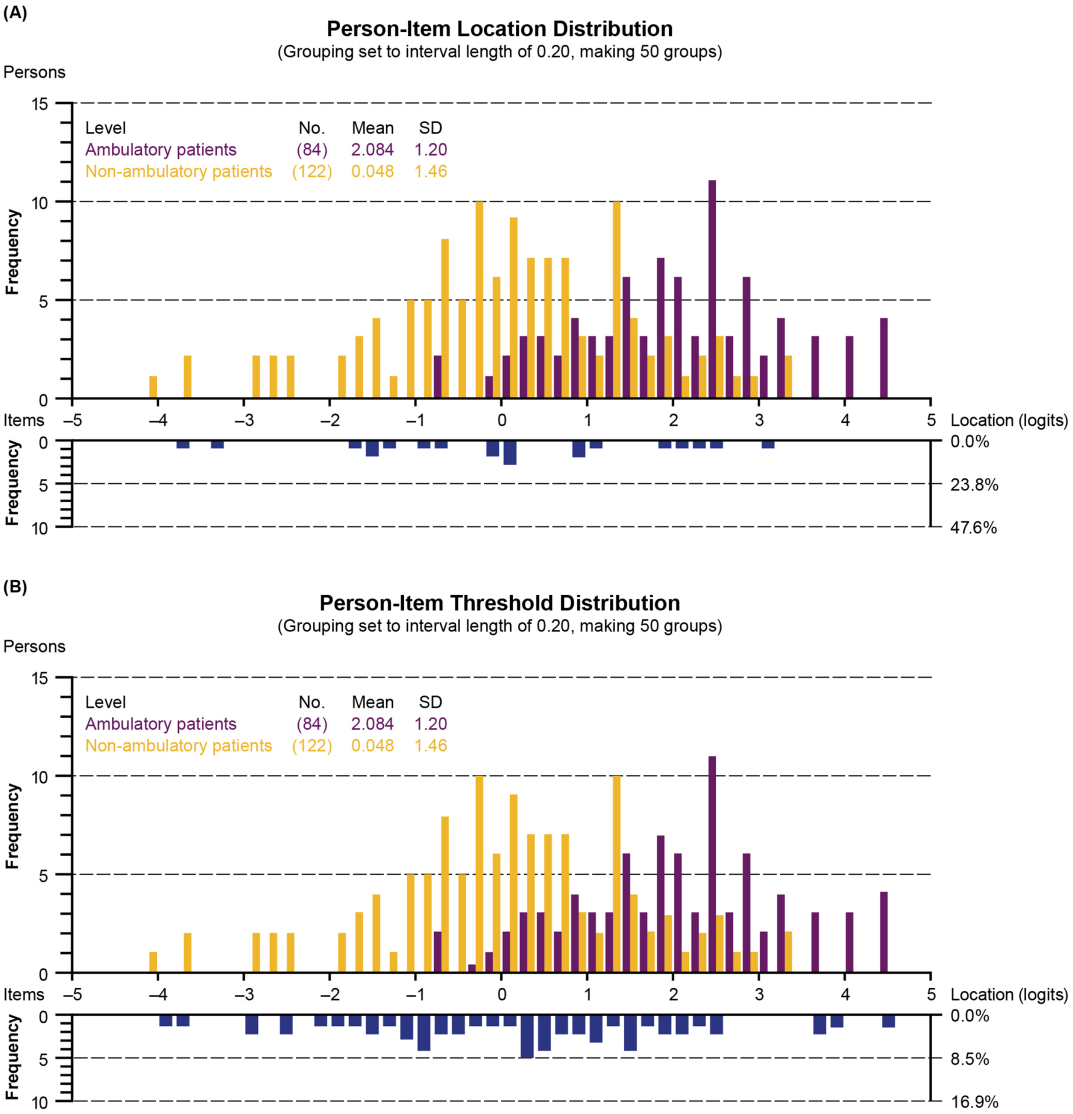
Person-item location **(A)** and threshold **(B)** distribution according to ambulatory status (grouping set to interval length of 0.20, making 50 groups). Children with DMD with the highest upper extremity level and the most difficult items are plotted on the right side of the histogram. Those with the lowest upper extremity level and the least difficult items are plotted on the left side. SD, standard deviation.

An association between ambulatory status and upper extremity function was observed, as illustrated by the person-item distribution plots broken down by ambulatory status (Figure 7). The average person-item location on the upper extremity function continuum estimated by the Rasch model also reflects the difference between groups, with an average of 2.08 for ambulatory participants versus 0.05 for non-ambulatory participants. However, no significant DIF was identified for the ambulatory status of the participants in this analysis, as none of the 42 *p* values (2 per item, one assessing the significance of uniform DIF and one assessing nonuniform DIF) were significant at the 0.05 threshold after application of the Bonferroni correction.

## 4 Discussion

To the best of our knowledge, this is the first study to evaluate the validity and psychometric performance of the PROMIS PP UE item bank in children with DMD using a Rasch analysis. The customized 21 items from the upper extremity function item bank achieved good overall model fit, with all items and persons showing good fit to Rasch model expectations. Some residual correlations were observed between items; however, despite the residual correlation, retaining them was justified because the items in question address different aspects of the underlying construct. The items that were easiest to perform were “Lift a cup to drink,” “Use mouse/touchpad on computer,” and “Brush teeth,” and the most difficult items appeared to be “Pull open heavy doors” and “Need help with bath.” Overall, the item ordering is consistent with progression of weakness and natural history of functional decline in late ambulatory and non-ambulatory children with DMD. Therefore, this alignment reflects the pathophysiology of DMD, characterized by a well-established pattern of progressive loss of muscle function and strength over time (3).

The psychometric analysis indicates that the customized measure is effective in measuring upper extremity function in individuals with DMD ages 8 years and older across most levels of function, but this measure may not be as effective for those with the highest levels of function based on person-item threshold distribution. In fact, the only item in a high position was the ability to open heavy doors. However, this item lacks clarity regarding the definition of a heavy door, which could lead to varied responses from ambulatory and non-ambulatory participants because of factors unrelated to upper extremity function, such as difficulty gaining adequate access to the door in a wheelchair. Therefore, this finding highlights the necessity for additional assessment of the content validity of this item specifically for DMD. It also calls for further evaluation of conceptual coverage targeting patients with higher levels of upper extremity function.

The published literature on PROMIS measures in DMD is limited, which precludes a comparison of our results with directly relevant literature. The Performance of Upper Limb (PUL) module is a widely used instrument to measure upper limb function in clinical and research settings, but it relies on clinician ratings rather than patient- or caregiver-reported outcomes (9, 28). A few PRO instruments are available that assess upper limb function specifically in late ambulatory and non-ambulatory children with DMD, such as the Patient-Reported Outcome Measure for the Upper Limb (PROM UL) (28). The PROM UL addresses a broad range of symptoms and includes elements of activities of daily living. Similarly, it can also be completed by a caregiver, as with the PROMIS PP UE item bank (28). However, the PROM UL was developed more recently and may require further evaluation of its reliability over time and its responsiveness to detect disease progression (9, 28). The ACTIVLIM is a patient- or observer-reported assessment tool designed to evaluate activity limitations in individuals with upper and/or lower limb impairments. It specifically measures an individual’s ability to perform daily activities (29). Additionally, The Disabilities of the Arm, Shoulder, and Hand Questionnaire (DASH) is another measure designed to assess levels of disability in the upper extremity (30). However, there is currently no evidence in the literature indicating its specific use in DMD. The Upper Limb Short Questionnaire (ULSQ) assesses upper limb function, pain, and stiffness (31). Overall, PROMIS instruments have been a National Institutes of Health–funded resource for clinical researchers for nearly 20 years and have been shown to capture disease progression (32–35). Establishing PROMIS PP UE construct validity against PUL can further verify its generalizability across individuals with DMD at various stages of disease progression.

In the present study, all children had a confirmed genetic diagnosis consistent with DMD. The large number of PROMIS observations included in this study permitted conduct of the Rasch psychometric analysis of a generic ObsRO item bank, ensuring that children with DMD who were ambulatory or non-ambulatory had an accurate assessment of upper extremity function. PRO/ObsRO measures could be used in clinical research more easily if they included a reliable scoring system and an item bank that was appropriate for the intended use (36). Emphasis on caregiver assessment makes it possible to obtain information about the daily functioning, abilities, and difficulties of children with DMD in a consistent manner over time (11). For a rare disease such as DMD, the number of observations that were available for the analysis constituted a satisfactory sample. These findings are pertinent considering the dearth of reliable, content-valid PRO/ObsRO measures in DMD clinical research. Finally, the measure focused on upper extremity function, which is crucial for understanding the progression of the disease, especially in non-ambulatory individuals (5).

### 4.1 Limitations

This study has some potential limitations. Although the UE items were not discussed with parents or patients during clinic visits, efforts are being made to establish the content validity of these items. A separate cognitive debrief study is currently underway to evaluate and confirm their relevance and comprehensiveness. Additionally, this analysis does not provide information about suitability of UE items and the overall scale in a younger age group, as the study’s lower age limit was 8. Therefore, investigation of limitations in upper limb function in daily life of children younger than 8 years of age warrants further investigation. Few of the included patients were at the highest or lowest end of the spectrum of upper extremity function. It would be beneficial to have had more patients with lower levels of functioning to more effectively examine the items’ capabilities in measuring difficulties with upper extremity function. Regardless, discriminative power was sufficient. Future research should also evaluate whether the scale may benefit from supplementation with additional items to target the higher end of the upper extremity function in DMD. Due to the rare nature of DMD and the fact that each participant received specialized care at the same neuromuscular clinic, sociodemographic information was excluded to avoid unintentional identification of participants. Despite our study having a sufficient sample size, especially for a rare disease such as DMD, the sample may not be truly representative of the DMD population as a whole. Given that this is the first study to publish PROMIS PP UE data in DMD, testing for convergent/divergent validity and employing both classical and modern test theories may be appropriate for discerning psychometric findings. Lastly, considering that changes in DMD appear to occur gradually over time, conducting longitudinal data analysis is essential; this will help assess the stability of the scale over time and its sensitivity in capturing disease progression.

The insights gleaned from this study will have meaningful implications for both clinical practice and research. Patient-reported or caregiver-reported disease impact also plays a crucial role in supporting drug registration, particularly for slowly progressive conditions like DMD. The practical utility of a robust measure is therefore essential for ensuring accurate assessments and improving outcomes measurement in the relevant field. Ensuring that the measure is not only psychometrically valid but also practical and feasible to implement enhances its value in evaluating disease progression, treatment response, and patient outcomes. The PROMIS PP UE measure may thus help to direct clinical care and enable proactive planning for the changing needs of children with DMD, in addition to serving as a sensitive measure facilitating therapeutic development in DMD.

In conclusion, this customized, 21-item PROMIS PP UE measure fits the Rasch model and was able to differentiate between respondents with varying upper extremity function levels. The results of this study indicate that the customized PROMIS PP UE item measure may serve as a reliable option for assessing upper extremity function in DMD from a caregiver’s perspective.

## Data Availability

Qualified researchers may request access to the data that support the findings of this study from Sarepta Therapeutics, Inc., by contacting medinfo@sarepta.com.

## Footnotes

^1^ https://www.promishealth.org/57461-2/

## References

[1] Szabo SM Salhany RM Deighton A Harwood M Mah J Gooch KL. The clinical course of Duchenne muscular dystrophy in the corticosteroid treatment era: a systematic literature review. Orphanet J Rare Dis. (2021) 16:237. doi: 10.1186/s13023-021-01862-w, PMID: 34022943 PMC8141220

[2] Szabo SM Audhya IF Malone DC Feeny D Gooch KL. Characterizing health state utilities associated with Duchenne muscular dystrophy: a systematic review. Qual Life Res. (2020) 29:593–605. doi: 10.1007/s11136-019-02355-x, PMID: 31811595 PMC7028804

[3] Powell PA Carlton J Woods HB Mazzone P. Measuring quality of life in Duchenne muscular dystrophy: a systematic review of the content and structural validity of commonly used instruments. Health Qual Life Outcomes. (2020) 18:263. doi: 10.1186/s12955-020-01511-z, PMID: 32746836 PMC7397669

[4] Crisafulli S Sultana J Fontana A Salvo F Messina S Trifirò G. Global epidemiology of Duchenne muscular dystrophy: an updated systematic review and meta-analysis. Orphanet J Rare Dis. (2020) 15:141. doi: 10.1186/s13023-020-01430-8, PMID: 32503598 PMC7275323

[5] Janssen MM Hendriks JC Geurts AC de Groot IJ. Variables associated with upper extremity function in patients with Duchenne muscular dystrophy. J Neurol. (2016) 263:1810–8. doi: 10.1007/s00415-016-8193-1, PMID: 27314968 PMC5010825

[6] Mendell JR Khan N Sha N Eliopoulos H McDonald CM Goemans N. Comparison of long-term ambulatory function in patients with Duchenne muscular dystrophy treated with eteplirsen and matched natural history controls. J Neuromuscul Dis. (2021) 8:469–79. doi: 10.3233/jnd-200548, PMID: 33523015 PMC8385516

[7] Ryder S Leadley RM Armstrong N Westwood M de Kock S Butt T. The burden, epidemiology, costs and treatment for Duchenne muscular dystrophy: an evidence review. Orphanet J Rare Dis. (2017) 12:79. doi: 10.1186/s13023-017-0631-3, PMID: 28446219 PMC5405509

[8] Brown V Merikle E Johnston K Gooch K Audhya I Lowes L. A qualitative study to understand the Duchenne muscular dystrophy experience from the parent/patient perspective. J Patient Rep Outcomes. (2023) 7:129. doi: 10.1186/s41687-023-00669-6, PMID: 38085412 PMC10716079

[9] Klingels K Mayhew AG Mazzone ES Duong T Decostre V Werlauff U. Development of a patient-reported outcome measure for upper limb function in Duchenne muscular dystrophy: DMD upper limb PROM. Dev Med Child Neurol. (2017) 59:224–31. doi: 10.1111/dmcn.13277, PMID: 27671699

[10] Slade A Isa F Kyte D Pankhurst T Kerecuk L Ferguson J. Patient reported outcome measures in rare diseases: a narrative review. Orphanet J Rare Dis. (2018) 13:61. doi: 10.1186/s13023-018-0810-x, PMID: 29688860 PMC5914068

[11] Schwartz CE Stark RB Cella D Borowiec K Gooch KL Audhya IF. Measuring Duchenne muscular dystrophy impact: development of a proxy-reported measure derived from PROMIS item banks. Orphanet J Rare Dis. (2021) 16:487. doi: 10.1186/s13023-021-02114-7, PMID: 34809687 PMC8607700

[12] Nelson EC Eftimovska E Lind C Hager A Wasson JH Lindblad S. Patient reported outcome measures in practice. BMJ. (2015) 350:g7818. doi: 10.1136/bmj.g7818, PMID: 25670183

[13] Uttley L Carlton J Woods HB Brazier J. A review of quality of life themes in Duchenne muscular dystrophy for patients and carers. Health Qual Life Outcomes. (2018) 16:237. doi: 10.1186/s12955-018-1062-0, PMID: 30567556 PMC6299926

[14] Powell PA Carlton J Rowen D Chandler F Guglieri M Brazier JE. Development of a new quality of life measure for Duchenne muscular dystrophy using mixed methods: the DMD-QoL. Neurology. (2021) 96:e2438–50. doi: 10.1212/wnl.0000000000011896, PMID: 33785551 PMC8166440

[15] Rowen D Powell P Mukuria C Carlton J Norman R Brazier J. Deriving a preference-based measure for people with Duchenne muscular dystrophy from the DMD-QoL. Value Health. (2021) 24:1499–510. doi: 10.1016/j.jval.2021.03.007, PMID: 34593174

[16] Uzark K King E Cripe L Spicer R Sage J Kinnett K. Health-related quality of life in children and adolescents with Duchenne muscular dystrophy. Pediatrics. (2012) 130:e1559–66. doi: 10.1542/peds.2012-0858, PMID: 23129083

[17] Schwartz CE Stark RB Borowiec K Audhya IF Gooch KL. Interplay of disability, caregiver impact, and out-of-pocket expenditures in Duchenne muscular dystrophy: a cohort study. J Patient Rep Outcomes. (2022) 6:21. doi: 10.1186/s41687-022-00425-2, PMID: 35267108 PMC8908951

[18] Lowes LP Le Reun CM Alfano LN Reash NF Iammarino MA Patel S. Psychometric evaluation of the PROMIS parent proxy mobility item bank for use in Duchenne muscular dystrophy. Dev Med Child Neurol. (2024) 00:1–12. doi: 10.1111/dmcn.16198, PMID: 39697056 PMC12134438

[19] Mendell JR Muntoni F McDonald CM Mercuri EM Ciafaloni E Komaki H. AAV gene therapy for Duchenne muscular dystrophy: the EMBARK phase 3 randomized trial. Nat Med. (2024) 31:332–41. doi: 10.1038/s41591-024-03304-z, PMID: 39385046 PMC11750718

[20] Galloway H Newman E. Is there a difference between child self-ratings and parent proxy-ratings of the quality of life of children with a diagnosis of attention-deficit hyperactivity disorder (ADHD)? A systematic review of the literature. Attention Defic. Hyperact. Dis. (2017) 9:11–29. doi: 10.1007/s12402-016-0210-9, PMID: 28005216 PMC5323486

[21] Jiang M Ma Y Li M Meng R Ma A Chen P. A comparison of self-reported and proxy-reported health utilities in children: a systematic review and meta-analysis. Health Qual Life Outcomes. (2021) 19:45. doi: 10.1186/s12955-021-01677-0, PMID: 33546723 PMC7866432

[22] Bruce B Fries J Lingala B Hussain YN Krishnan E. Development and assessment of floor and ceiling items for the PROMIS physical function item bank. Arthritis Res Ther. (2013) 15:R144. doi: 10.1186/ar4327, PMID: 24286166 PMC3978724

[23] Krabbe PFM. Chapter 10—item response theory In: Krabbe PFM, editor. The measurement of health and health status. San Diego, California: Academic Press (2017). 171–95.

[24] Masters GN. A Rasch model for partial credit scoring. Psychometrika. (1982) 47:149–74. doi: 10.1007/BF02296272

[25] Smith EV. Detecting and evaluating the impact of multidimensionality using item fit statistics and principal component analysis of residuals. J Appl Meas. (2002) 3:205–31. PMID: 12011501

[26] Tennant A Pallant JF. Unidimensionality matters! (a tale of two Smiths?). Rasch Meas Trans. (2006) 20:1048–51.

[27] Tennant A Conaghan PG. The Rasch measurement model in rheumatology: what is it and why use it? When should it be applied, and what should one look for in a Rasch paper? Arthritis Rheum. (2007) 57:1358–62. doi: 10.1002/art.23108, PMID: 18050173

[28] Cicala G Pane M Coratti G Brogna C Fanelli L Norcia G. Patient reported outcome measure for upper limb in Duchenne muscular dystrophy: correlation with PUL2.0. Neuromuscul Disord. (2023) 33:69–73. doi: 10.1016/j.nmd.2023.07.003, PMID: 37612177

[29] Vandervelde L Van den Bergh PY Goemans N Thonnard JL. ACTIVLIM: a Rasch-built measure of activity limitations in children and adults with neuromuscular disorders. Neuromuscul Disord. (2007) 17:459–69. doi: 10.1016/j.nmd.2007.02.013, PMID: 17433675

[30] Dixon D Johnston M McQueen M Court-Brown C. The disabilities of the arm, shoulder and hand questionnaire (DASH) can measure the impairment, activity limitations and participation restriction constructs from the international classification of functioning, disability and health (ICF). BMC Musculoskelet Disord. (2008) 9:114. doi: 10.1186/1471-2474-9-114, PMID: 18715495 PMC2533660

[31] Choi YA Shin HI. Reliability and validity of upper limb short questionnaire for Duchenne muscular dystrophy. Disabil Rehabil. (2022) 44:2448–55. doi: 10.1080/09638288.2020.1829107, PMID: 33027595

[32] Cella D Riley W Stone A Rothrock N Reeve B Yount S. The patient-reported outcomes measurement information system (PROMIS) developed and tested its first wave of adult self-reported health outcome item banks: 2005-2008. J Clin Epidemiol. (2010) 63:1179–94. doi: 10.1016/j.jclinepi.2010.04.011, PMID: 20685078 PMC2965562

[33] Kaat AJ Buckenmaier CT 3rd Cook KF Rothrock NE Schalet BD Gershon RC. The expansion and validation of a new upper extremity item bank for the patient-reported outcomes measurement information system® (PROMIS). J Patient Rep Outcomes. (2019) 3:69. doi: 10.1186/s41687-019-0158-6, PMID: 31773413 PMC6879697

[34] Cook KF Jensen SE Schalet BD Beaumont JL Amtmann D Czajkowski S. PROMIS measures of pain, fatigue, negative affect, physical function, and social function demonstrated clinical validity across a range of chronic conditions. J Clin Epidemiol. (2016) 73:89–102. doi: 10.1016/j.jclinepi.2015.08.038, PMID: 26952842 PMC5131708

[35] Jensen RE Moinpour CM Potosky AL Lobo T Hahn EA Hays RD. Responsiveness of 8 patient-reported outcomes measurement information system (PROMIS) measures in a large, community-based cancer study cohort. Cancer. (2017) 123:327–35. doi: 10.1002/cncr.30354, PMID: 27696377 PMC5222745

[36] Whittal A Meregaglia M Nicod E. The use of patient-reported outcome measures in rare diseases and implications for health technology assessment. Patient. (2021) 14:485–503. doi: 10.1007/s40271-020-00493-w, PMID: 33462774 PMC8357707

