# Supplementary material for: Psychometric analysis of the patient-reported outcomes measurement information system parent proxy physical function–upper extremity item bank for children with Duchenne muscular dystrophy
Source: Front Neurol. 2025 May 13;16:1481825. doi: 10.3389/fneur.2025.1481825 (PMC12146635; doi:10.3389/fneur.2025.1481825)
Supplement: Supplementary file 1 [file Supplementary_file_1.docx]

**Supplementary Material**

**Figure S1. Rasch analysis flowchart**


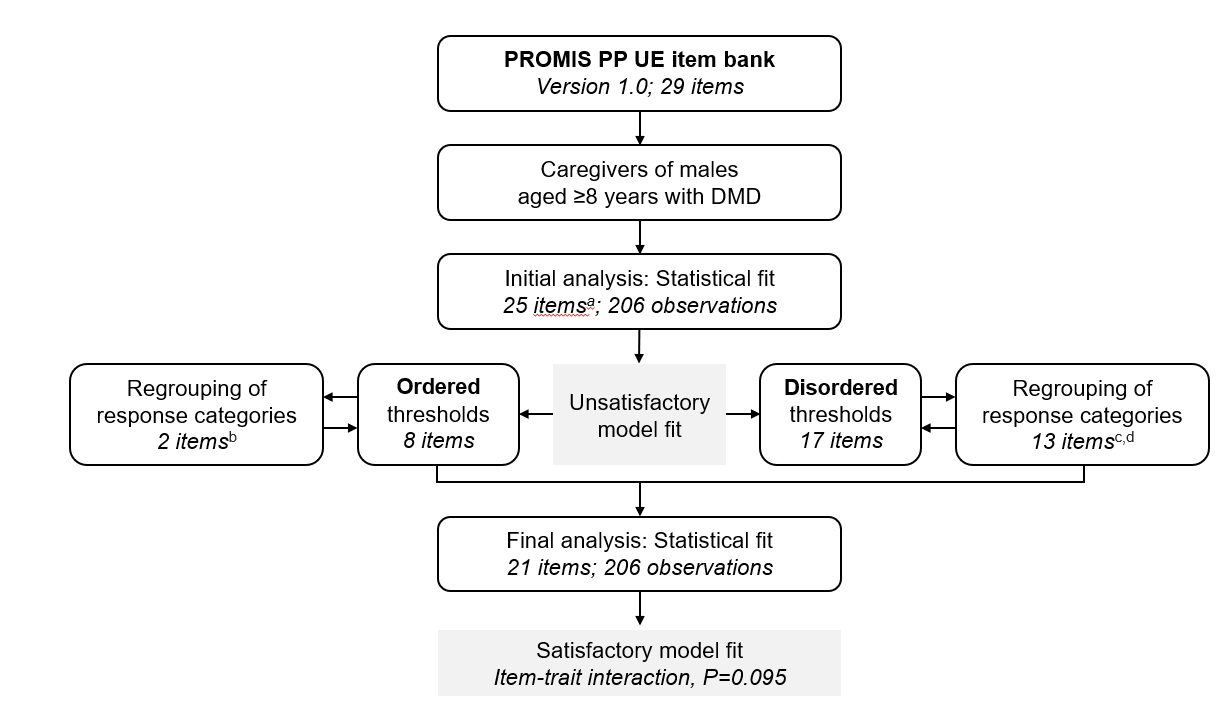
 ^a^Four of the 29 items included in the PROMIS PP UE item bank were excluded as they were deemed to be not clinically relevant or not applicable to pediatric children with DMD by neuromuscular physical therapists.

^b^Two of the initially ordered items were reorganized, owing to small occupancy of certain response categories.

^c^13 items with disordered thresholds were reorganized, based on mathematical results and clinical judgment.

^d^Four of the items with disordered thresholds were excluded, based on clinical judgment.

DMD, Duchenne muscular dystrophy; PP, Parent Proxy; PROMIS, Patient-Reported Outcomes Measurement Information System; UE, Upper Extremity.

**Table S1. Residual correlation matrix**

| **Item** | **I-01** | **I-02** | **I-04** | **I-05** | **I-06** | **I-08** | **I-09** | **I-10** | **I-12** | **I-13** | **I-14** | **I-15** | **I-16** | **I-17** | **I-18** | **I-19** | **I-21** | **I-22** | **I-26** | **I-27** | **I-28** |
| --- | --- | --- | --- | --- | --- | --- | --- | --- | --- | --- | --- | --- | --- | --- | --- | --- | --- | --- | --- | --- | --- |
| I-01 button shorts or pants | 1 |  |  |  |  |  |  |  |  |  |  |  |  |  |  |  |  |  |  |  |  |
| I-02 open a jar | −0.097 | 1 |  |  |  |  |  |  |  |  |  |  |  |  |  |  |  |  |  |  |  |
| I-04 pour drink from full pitcher | −0.148 | 0.088 | 1 |  |  |  |  |  |  |  |  |  |  |  |  |  |  |  |  |  |  |
| I-05 pull shirt over head | −0.120 | −0.081 | −0.057 | 1 |  |  |  |  |  |  |  |  |  |  |  |  |  |  |  |  |  |
| I-06 pull open heavy doors | −0.231 | 0.160 | 0.195 | −0.011 | 1 |  |  |  |  |  |  |  |  |  |  |  |  |  |  |  |  |
| I-08 use key to unlock door | −0.030 | −0.008 | −0.018 | −0.183 | −0.112 | 1 |  |  |  |  |  |  |  |  |  |  |  |  |  |  |  |
| I-09 zip up clothes | 0.195 | −0.291 | −0.106 | −.044 | −0.072 | −0.160 | 1 |  |  |  |  |  |  |  |  |  |  |  |  |  |  |
| I-10 put toothpaste on toothbrush | −0.024 | −0.082 | −0.046 | −0.023 | −0.209 | −0.022 | 0.060 | 1 |  |  |  |  |  |  |  |  |  |  |  |  |  |
| I-12 pull on and fasten seatbelt | 0.057 | −0.077 | −0.136 | −0.043 | −0.058 | −0.089 | 0.033 | 0.075 | 1 |  |  |  |  |  |  |  |  |  |  |  |  |
| I-13 put on socks | 0.003 | −0.309 | −0.187 | 0.162 | −0.021 | −0.079 | 0.350 | −0.098 | 0.125 | 1 |  |  |  |  |  |  |  |  |  |  |  |
| I-14 cut paper with scissors | −0.122 | 0.015 | −0.066 | −0.164 | −0.033 | 0.096 | −0.051 | −0.062 | −0.062 | −0.134 | 1 |  |  |  |  |  |  |  |  |  |  |
| I-15 open clothing drawers | −0.076 | −0.110 | −0.124 | −0.098 | 0.067 | −0.172 | 0.027 | −0.055 | 0.083 | 0.021 | −0.081 | 1 |  |  |  |  |  |  |  |  |  |
| I-16 hold a full cup | −0.151 | −0.066 | 0.126 | 0.167 | 0.047 | −0.230 | −0.185 | −0.019 | −0.119 | −0.155 | −0.008 | −0.031 | 1 |  |  |  |  |  |  |  |  |
| I-17 lift a cup to drink | -0.063 | 0.042 | 0.048 | 0.104 | 0.039 | 0.030 | 0.013 | 0.050 | 0.076 | 0.082 | -0.115 | -0.083 | 0.034 | 1 |  |  |  |  |  |  |  |
| I-18 use mouse on computer | 0.097 | −0.082 | −0.209 | 0.070 | −0.150 | 0.052 | −0.042 | 0.011 | −0.026 | 0.001 | −0.009 | −0.019 | −0.058 | −0.022 | 1 |  |  |  |  |  |  |
| I-19 wash face with a cloth | −0.171 | −0.176 | −0.084 | 0.014 | −0.120 | −0.044 | 0.031 | 0.136 | −0.077 | −0.100 | −0.084 | -0.041 | 0.039 | 0.068 | 0.023 | 1 |  |  |  |  |  |
| I-21 dry back with a towel | −0.052 | −0.061 | −0.259 | −0.062 | −0.054 | −0.149 | −0.038 | −0.103 | −0.026 | 0.163 | −0.172 | 0.096 | −0.217 | 0.042 | 0.028 | -0.073 | 1 |  |  |  |  |
| I-22 turn door handles | −0.164 | −0.032 | −0.015 | −0.281 | 0.021 | 0.192 | −0.210 | −0.075 | −0.053 | −0.183 | 0.188 | 0.026 | −0.121 | −0.122 | −0.048 | 0.019 | −0.043 | 1 |  |  |  |
| I-26 brush teeth | −0.067 | −0.039 | 0.028 | −0.146 | −0.093 | −0.089 | −0.112 | −0.032 | −0.034 | −0.166 | 0.028 | −0.044 | −0.035 | −0.110 | 0.093 | 0.245 | −0.014 | −0.017 | 1 |  |  |
| I-27 write with pen/pencil | 0.049 | 0.107 | −0.221 | −0.075 | −0.184 | 0.087 | −0.179 | −0.081 | −0.126 | −0.068 | 0.167 | −0.198 | −0.047 | −0.130 | 0.126 | -0.114 | 0.027 | −0.078 | 0.158 | 1 |  |
| I-28 need help with bath | −0.026 | −0.043 | −0.043 | −0.052 | 0.010 | −0.137 | 0.055 | 0.023 | −0.113 | 0.098 | −0.232 | −0.063 | −0.091 | −0.144 | −0.077 | -0.032 | 0.121 | −0.071 | −0.014 | 0.051 | 1 |
